# Being questioned and receiving advice about alcohol and smoking in health care: Associations with patients' characteristics, health behavior, and reported stage of change

**DOI:** 10.1186/1747-597X-5-30

**Published:** 2010-11-29

**Authors:** Kozma Ahacic, Peter Allebeck, Kerstin Damström Thakker

**Affiliations:** 1 Karolinska Institutet, Department of Public Health Sciences, Social medicine, Box 170 70, 104 62 Stockholm, Sweden; 2 Karolinska Institutet, Department of Public Health Sciences, Applied Public Health, Box 170 70, 104 62 Stockholm, Sweden

## Abstract

**Background:**

Alcohol habits are more rarely addressed than other health behavior topics in Swedish health care. This study examined whether differences between topics could be explained by their different associations with patient characteristics or by the differences in the prevalence of the disadvantageous health behavior, i.e., excessive alcohol use and smoking. The study moreover examined whether simply being asked questions about behavior, i.e., alcohol use or smoking, was associated with reported change.

**Methods:**

The study was based on a cross-sectional postal survey (n = 4 238, response rate 56.5 percent) representative of the adult population in Stockholm County in 2003. Retrospective self-reports were used to assess health care visits during the past 12 months, the questions and advice received there, patients characteristics, health behavior, and the present stage of change. Logistic regression analysis was used to estimate the associations among the 68 percent who had visited health care.

**Results:**

Among the health care visitors, 23 percent reported being asked about their alcohol habits, and 3 percent reported receiving advice or/and support to modify their alcohol use - fewer than for smoking, physical exercise, or diet. When regression models adjusted for patient characteristics, the differences between health behaviors in the extent of questioning and advice remained. However, when the models also adjusted for smoking and alcohol consumption there was no difference between smoking and alcohol-related advice. In fact one-third of the present smokers and two-fifths of the persons dependent on alcohol reported having receiving advice the previous 12 months. Those who reported being asked questions or receiving advice more often reported a decreased alcohol use and similarly intended to cease smoking within 6 months. Questions about alcohol use were moreover related to a later stage of stage of change independently of advice among women but not among men.

**Conclusions:**

While most patients are never addressed, many in the target groups seem to be reached anyway. Besides advice, already addressing alcohol habits appears to be associated with change. The results also indicate that gender possibly plays a role in the relationship between advice and the stage of change.

## Introduction

The effect of brief primary care counseling in connection with screening is widely recognized [1-3]. In Sweden there is no mandatory screening of alcohol use in primary care and presently only a minority of the visitors is questioned about their health behavior, i.e., physical exercise, alcohol, dietary habits, or smoking. Even fewer receive subsequent life style advice and alcohol seems to be addressed less often than the other health behavior topics. A survey of 41 primary care centers in a rural area in the south of Sweden examined how many patients reported having received health behavior advice. The proportion of primary care patients who had received advice related to alcohol was 5 percent, while corresponding estimates for smoking was 9 percent, physical exercise 16 percent, and diet 13 percent [4]. Another study addressing all general practitioners and nurses working in primary health care in Sweden asked how frequently they addressed drinking and other lifestyle issues with their patients [5]. This study similarly found that drinking was less often addressed in comparison to smoking, overweight, exercise, and stress.

Whether health behaviors are addressed or not seems to be related to patient characteristics, such as gender, age, or health status [4]. While many studies have examined the reasons for not addressing alcohol behavior [5-11], it has not been examined whether different patient characteristics might explain why alcohol is more rarely addressed than smoking, physical exercise, or dietary habits. The fact that there are fewer excessive drinkers than smokers could also explain why alcohol is more rarely addressed than smoking, something which has not been examined before. Possibly because of the apparent benefits of mandatory screening [12], little attention has focused on to which extent target groups have been reached.

In Sweden a limit for low risk drinking is considered to be a maximum of 14 drinks per week for men and 9 for women [13]. Hazardous use of alcohol is usually identified by screening instruments, such as AUDIT (the Alcohol Use Disorders Identification Test), measuring both alcohol consumption and its consequences on the individual's physical and mental health [14]. Numerous behavioral, cognitive, and physiological symptoms indicate the more severe state, alcohol dependence. In one study fourteen percent of the general population in Sweden was estimated to have a hazardous alcohol use i.e., an AUDIT-score 8-15 for men and 6-13 for women, and three percent were indicated to have an alcohol dependence, i.e., an AUDIT-score 15+ for men and 13+ for women [15]. In comparison, a fifth of the population has been estimated to be daily smokers [16].

According to the AUDIT manual abstainers or non-hazardous alcohol users should also be informed about the health consequences of alcohol consumption to increase the general population's awareness when screening for alcohol use [17,18]. While simple advice may be sufficient for hazardous users, the manual recommends advice and brief counseling, monitoring or even treatment by specialists for those dependent on alcohol.

While brief counseling in primary care is an effective means for achieving improvements in individuals' health behavior [1,19-22], it has been less clear whether the less structured advice given in ordinary primary care settings [23] or whether already simply addressing health behaviors, such as alcohol use, [24] might be associated with reported change. Effects similar to that of brief counseling have been indicated for simply screening of alcohol use [25,26]. To our knowledge this is the first study to that examines whether the reported talk about tobacco or alcohol at a previous ordinary health care visit might be related to the stage of change regarding smoking cessation or alcohol use in a cross-sectional population sample. The readiness to change alcohol habits did increase after consultations in a cohort of excessive drinking patients followed for six months [27]. While mechanisms underlying change remain unclear, talking to primary health care personal may, for example, shed light on the pros and cons of the relevant behavior which may facilitate behavioral change [28].

This study analyzes the extent of alcohol related advice in relation to some of its correlates among the general population visiting health care in Stockholm County. It asks: a) Was alcohol consumption addressed less often than other health areas; b) Could different patient characteristics or smoking and alcohol habits explain why alcohol is more rarely addressed than smoking; c) Were questions and advice associated with reports of decreased alcohol use or smoking cessation; d) Were questions about alcohol related to reported change independently of advice?

## Methods

### Sampling frame

Based population census a cross-sectional randomized sample n = 7500 representative of the adult population aged 18 to 70 years in Stockholm County in Sweden was surveyed in October 2003 using a comprehensive postal questionnaire. The response rate was 56.5 percent (n = 4 238). Of the responders, 68.4 percent (n = 2 786) reported having visited primary health care during the last 12 months (internal missing values; n = 164).

### Measures

#### Selection variables

The selection of the primary care visitors among the respondents was based on the question: "Have you at any time during the last 12 months visited or been treated by a physician or any other health care personnel in primary care? The question does not refer to treatment in connection to inpatient hospital care". The response alternatives were: "1. Yes once"; "2. Yes, twice"; "3. Yes, three times or more"; or "4. No" for three different specified options of health care: "At primary care, a private or county physician reception"; "At emergence care reception in a hospital"; and "Other kind of health care (not inpatient care at a hospital)". These answers were then collapsed; while those with any form of yes equaling one or more health care visits were selected for the study's analyses, those with no health care visits were disregarded.

#### Independent variables

The independent variables used in the analysis were gender, age, social class, sick-leave, physical and mental health, smoking/snuff, and alcohol use. The gender distributions as well as other independent variables are presented in Table 1. Age was divided into ten year age groups and coded as a categorical variable. The variable social class is based on a classification scheme which groups the respondents' occupations on basis of their relation to the process of production and their position in the labor market [29]. This classification scheme has customary been applied in the Swedish context and the variable here differentiates between three levels of white-collar workers and manual workers. The question concerning sick-leave: "How many days have you altogether been absent from work due to sick-leave within the past 12 months?" could be answered with one out the five pre-coded response alternatives presented in Table 1. Similarly, the five possible response alternatives to the two self-reported health questions are also found in Table 1. The questions were; "How do you judge your physical and mental health condition to be at present? My physical health condition is..."; and "My mental health condition is...".

**Table 1:** Distributions of background variables in the sample and among health care visitors

	In the whole sample		Among health care visitors	
**Variables categories**	**%**	**n**	**%**	**n**

**Gender:**				
Men	44.3	*1877*	40.7***	*1135*
Women	55.7	*2361*	59.3***	*1651*
Total	100.0	*4238*	100.0	*2786*
				
**Age:**				
*average age and age span:*	*43.0 years*	*18-70 years*	*43.4 years*	*18-70 years*
**Age group:**				
18-24	10.1	*428*	10.2	*283*
25-39	22.8	*965*	22.6	*630*
35-44	22.2	*941*	20.9**	*583*
45-54	18.6	*787*	18.2	*506*
55-64	19.9	*842*	21.2**	*591*
65-70	6.5	*275*	6.9	*193*
				
**Social class:**				
blue collar	21.6	*915*	21.9	*610*
lower white collar	15.2	*644*	16.2*	*451*
middle white collar	25.0	*1060*	25.1	*700*
higher white collar	24.5	*1037*	23.4*	*653*
missing values	13.7	*582*	13.4	*372*
				
**Sick-leave the last 12 months**				
None	41.6	*1764*	34.3***	*955*
1-7 days	35.2	*1490*	36.1	*1005*
8-30 days	11.0	*464*	14.3***	*399*
31-90 days	3.3	*138*	4.3***	*121*
90+ days	5.5	*233*	7.8***	*216*
missing values	3.5	*149*	3.2	*90*
				
**Physical health:**				
very good	38.1	*1613*	32.0***	*898*
Good	44.0	*1867*	46.8***	*1303*
neither good or bad	11.0	*466*	12.7***	*353*
bad or very bad	5.6	*238*	7.9***	*220*
missing values	1.3	*54*	0.6***	*17*
				
**Mental health:**				
very good	46.0	*1949*	41.8***	*1165*
Good	37.1	*1570*	39.6***	*1103*
neither good or bad	9.4	*404*	10.8***	*301*
bad or very bad	5.2	*219*	6.3***	*175*
missing values	2.3	*96*	1.5***	*42*
				
**Smoking:**				
never smoked	48.0	*2033*	46.3**	*1289*
present smoker	20.3	*860*	20.8	*579*
earlier smoker	30.3	*1286*	31.6*	*881*
missing values	1.4	*59*	1.3	*37*
				
**Alcohol:**				
don't drink	7.6	*323*	7.5	*209*
non-hazardous use	78.9	*3343*	78.7	*2192*
hazardous use	10.5	*446*	10.9	*303*
dependent on alcohol	2.4	*102*	2.4	*67*
missing values	0.6	*24*	0.5	*15*

* p < 0.05 ** p < 0.01 *** p < 0.001 Significance levels are given for health care visitors in comparison to non-visitors using Wald chi-square tests in logistic regression models.

For smoking/snuff and alcohol use the questionnaire also included items directed at approximating the prevalence and frequency of the concerned health behavior. Smoking behavior was based on two items: "Have you ever smoked for more than a year (daily or now and then)? Yes/no" and "Do you smoke presently? Yes, daily/Yes, now and then/No, I've stopped". For alcohol use, the screening instrument AUDIT together with recommended cut offs were applied [30,31]. It is an instrument that has been translated and used in Swedish contexts [15,31-33]. It is composed of 10 items, referring to three domains. The first 3 items address frequency of drinking, typical quantity, and frequency of heavy drinking and reflect "hazardous use of alcohol". The next 3 items, "dependence symptoms" concern impaired control over drinking, increased salience of drinking, and morning drinking. The last 4 items, "harmful alcohol use" concern guilt after drinking, blackouts, alcohol related injuries, and whether others are concerned. The response of each item is given a score of 0-4 points and the total score spans from 0 to 40 points. Higher scores indicate a more hazardous use and recommended cut offs have been 8 points for men and 6 for women [34]. To differentiate between hazardous users and alcohol dependent people the higher alternative cut offs were used, 15 points for men and 13 for women [32].

#### Outcome variables

The studied outcomes concerned whether the respondents had been asked questions, received advice or help from primary health care during the last 12 months regarding dietary habits, physical exercise, smoking/snuff use, and alcohol use. The participants were asked whether they had been asked any questions about their health behavior, if they had received any advice concerning changing their behavior, and finally whether they had received any help to do so. The three questions were in the following form; "Has a physician or any other health care professional during the last 12 months asked questions about your health behavior?" and each could be answered yes or no for each of the four specified health behaviors. Due to the small number of participants who answered yes, advice and help were collapsed in the analysis.

The Transtheoretical Model (TTM) is a general model of intentional behavioral change [35]. TTM focuses on the decision making of the individual and defines the stage of change he is in [36,37]. The model sees change as gradually occurring through five different stages, ranging from precontemplation to maintenance; *precontemplation *- not thinking of changing the behavior; *contemplation *- considering a behavioral change but have not yet made a commitment; *preparation *- made decisions to change the behavior within a given period (30 days); *action *- having changed the behavior within the past 6 months; *maintenance *- sustaining the behavior change for at least 6 months. An item to capture the relevant stage of change was developed based on the stages indicated by TTM. Similar, so called algorithmic (see Migealt et al, 2005) approaches have been applied elsewhere (Belding et al, 1995; Migneault et al, 1997). The stage of change was measured using the question "Which of the following statements describes your situation best? Only one alternative may be chosen". Then six optional statements were given; a) I reduced my alcohol consumption more the 12 months ago; b) I reduced my alcohol consumption 6-12 months ago; c) I reduced my alcohol consumption less than 6 months ago; d) I intend to reduce my alcohol consumption within 30 days; e) I intend to reduce my alcohol consumption within 6 months; f) I do not intend to reduce my alcohol consumption within 6 months. Similar alternatives were used to measure a cessation of smoking. The first two alternatives a) and b) represented an alteration introduced to target the 12 months' time span used for the other dependent variables. In the study stage of change is viewed as an outcome and focus is on change rather than on the different stages. The customary etiquettes of the stages (precontemplation, contemplation, action etc) have not been applied since we felt that the retrospectively reported changes implied between the stages of change in the measure were better demonstrated if the wording in the operationalizations was kept.

### Statistical analysis

To present differences between the visitors and non-visitors with regards to the distribution of the independent variables significance levels were given for health care visitors in comparison to non-visitors in Table 1. For this comparison logistic regressions modeling the relevant outcomes were computed using SAS software. Both in these analyses and later, all significance levels come from Wald chi-square tests using logistic regressions.

#### Aims a) and b)

To determine whether alcohol use was less commonly addressed than other health behaviors, a series of logistic regressions were conducted for each health behavior. The dependent variable was being asked or not being asked questions about the particular health behavior and the independent variables were gender, age, socioeconomic position, sick-leave, physical and mental health, smoking and alcohol use. Another series of logistic regressions regarding receiving or not receiving advice/help were performed in the same manner. Independent variables were effect coded, i.e., each category was compared to the average of all categories rather than to the odds of a reference category. All bivariate associations were first examined and the results are presented in Table 2. The independent variables were then introduced in groups in multiple models: first models with no independent variables were estimated, then gender and age variables were added to each model, next socio-economic position and sick leave, then self-rated physical and mental health, and finally smoking and alcohol consumption variables. These multiple models' estimated odds for the intercept were recalculated into prevalence estimates and presented in figures, to make the results easily interpretable and comparable to previous studies. For comparisons between the different health behaviors, the 95 percent confidence intervals were checked. The McKelvey and Zavoina pseudo R2 gives a fair approximation of the explained variance [38].

**Table 2:** Percent who were a) questioned and b) advised/helped concerning their different health behaviors when visiting health care by the different background variables.

	a) Questioned				b) Advised or helped			
	Dietary habits	Physical exercise	Smoking or snuff use	Alcohol use	Dietary habits	Physical exercise	Smoking or snuff use	Alcohol use
**Total**								
	27.1	33.4	32.3	23.0	14.3	16.2	8.8	2.7
								
**Gender:**								
Men	30.7***	38.2***	33.2	28.1***	18.2***	18.9**	8.3	4.8***
Women	24.7***	30.2***	31.8	19.4***	11.6***	14.3**	9.1	1.3***
								
**Age group:**								
18-24	20.1*	20.7***	32.0	18.0	11.7	10.6*	10.6	2.2
25-34	28.8	32.7	37.8***	27.2***	10.4**	11.7*	6.8	2.2
35-44	25.2	33.8	29.3	22.7	12.8	16.6	7.0	2.6
45-54	29.7	39.4***	35.1*	25.2*	15.5	22.2***	10.3	2.4
55-64	29.9*	37.3**	31.6	22.4	18.8**	19.5**	11.7**	3.8
65-70	23.2	25.6	18.0***	12.4**	19.6*	12.6	6.0	3.6
								
**Social class:**								
blue-collar	26.2	31.3	34.2	23.0	18.6**	21.4***	15.8***	5.1***
lower white collar	25.5	32.7	31.5	21.2	12.4	13.4	9.0	2.0
middle white collar	27.0	32.8	32.2	22.3	13.2	16.2	7.8	1.7
higher white collar	29.9	38.7***	33.0	26.2*	12.7	13.4	4.8***	1.6
missing values	26.1	29.2	29.3	20.5	14.9	16.2	6.3	3.9
								
**Sick-leave:**								
None	24.4**	30.3***	27.1***	20.0**	12.6***	13.0***	5.8***	2.2
1-7 days	24.3**	28.7***	30.1**	20.9**	10.4***	10.9***	7.2**	2.0*
8-30 days	28.3	36.5	40.8*	23.9	15.3	20.9	14.2*	2.2
31-90 days	42.9***	48.3**	49.6***	35.1**	31.6***	33.6***	15.7	4.8
90+ days	45.3***	61.5***	45.5**	40.8***	28.6***	37.3***	15.8*	8.4***
missing value	19.1*	20.7***	23.5**	16.2*	15.8	16.7	10.3	3.8
								
**Physical health:**								
very good	21.8***	26.2***	29.3*	22.3*	8.4***	7.8***	5.2***	2.0*
good	26.4***	32.9**	31.1	21.1**	13.5	15.1**	8.3*	2.3
neither good or bad	35.4	44.9	38.2	26.4	23.3*	30.2**	13.7	2.9
bad or very bad	38.4	46.5	42.8	30.2	30.4***	35.2***	19.7**	8.5***
missing value	56.2*	64.3*	40.0	46.2	15.4	30.8	16.7	0^1^
								
**Mental health:**								
very good	24.5***	29.4***	30.9	21.7*	12.3	12.9	6.9	1.6
good	25.1***	33.3**	31.8	21.2**	13.2	15.3	9.0	2.3
neither good or bad	36.5	38.1	35.3	26.3	19.3	22.1	11.3	5.2
bad or very bad	39.8*	49.7**	40.5	35.2*	25.2	31.2	13.8	7.8
missing values	35.9	48.6	34.4	30.3	22.2	28.6	20.0	9.4
								
**Smoking:**								
never	26.5	32.7	26.8***	22.0	12.2*	13.3**	0.7***	1.4*
presently	28.6	33.2	47.1***	24.4	17.5	18.2	32.5***	5.7*
earlier	26.8	34.4	30.6	23.5	15.2	18.8	4.5	2.8
missing values	37.1	37.1	34.4	20.7	20.6	23.5	13.3	3.4
								
**Alcohol:**								
don't drink	27.0	30.8	31.0	23.4	17.8	21.6	7.8*	1.8*
non-hazardous use	26.7	33.8	31.3	21.8	13.7*	15.4*	7.8***	1.0***
hazardous use	29.0	31.4	38.5	26.5	15.2	16.3	14.0	7.3
dependent on alcohol	35.6	36.2	41.9	45.3***	22.0	24.1	22.0**	39.1***
missing values	15.4	45.4	27.3	10.0	0^2^	27.3	0^2^	0^2^
*Internal missing n*	*115*	*150*	*166*	*203*	*118*	*169*	*216*	*255*

Bivariate associations (n = 2786).

* p < 0.05 ** p < 0.01 *** p < 0.001 The significance level is given for each category contrasted against the average of the variable's all categories using Wald chi-square tests in logistic regression models. ^1 ^In estimation of significance levels for this model the category for "missing values" was collapsed with the category "very good". If this category is excluded instead the significance levels remain similar for the other categories. ^2 ^Category was collapsed with non-hazardous use in estimations. If this category is excluded significance levels remain similar.

#### Aim c)

For the next aim, i.e., to determine whether persons in different stages of change had received more or less questions about their alcohol, a bivariate logistic regression model was used. The dependent variable was being asked questions or not being asked questions about alcohol and the independent variable was stage of change. The stages of change were modeled with regular dummy variables, and the reference category was people who did not intend to reduce their alcohol consumption. Respondents who drank less than one glass of alcohol during the last twelve months were not addressed by this stage of change item and therefore excluded from modeling. Next, the having received advice/help was used as the dependent variable, with stages of change as independent variable. In an additional comparison for alcohol related advice the first three categories were collapsed and used as the reference category against which the other categories were contrasted. This was done in order to be able to compare the odds for those who had decreased their consumption against the odds for all those who had not or only intended to decrease their consumption. Two bivariate models, similar to the first two models for alcohol, also examined smoking. People who had never smoked were not addressed by the stage of change item and were therefore excluded from modeling. For alcohol the model concerned a reported decrease in use, while for tobacco it concerned a cessation of smoking.

#### Aim d)

To examine the last aim, whether questions about alcohol were related to reported change independently of advice, both questions and advice were entered together in a model, where the stage of change was the dependent variable. Questions and advice were independent variables and ordered logistic regressions were applied which modeled a later stage of change, using five stages of change as outcome. Since we were only interested in health advice given within the past year, people in the sixth stage of change, a decrease of alcohol consumption more than a year ago, were excluded. Besides questions and advice concerning alcohol habits, this analysis also used gender and age as independent variables (using continuous linear assumption for age, modeling the change of odds for each year of increase in age). In addition to the main effects, the models also included some of the second order interaction terms between the variables as they were significant and relevant for interpretations.

## Results

The distribution of the background variables in the whole sample indicated a slight biased response, Table 1. For example, the percentage of women in the whole sample, 56 percent, is somewhat higher than in the population and proportion white collar workers probably likewise.

### Characteristics of the heath care visitors

Table 1 also shows that women were overrepresented among the health care visitors. A lower percent with the best health self-ratings and the least sick-leave is also found among the health care visitors, while age, social class, smoking and alcohol use variables indicate no or small differences. Twenty-one percent were present smokers, eleven percent had a hazardous alcohol use, and two percent were indicated to be dependent on alcohol.

### Characteristics of the visitors receiving questions and advice

Table 2 shows that male health care visitors reported receiving significantly more questions and advice/help than female visitors concerning dietary habits, physical exercise, and alcohol use. Age patterns were diverse. Higher white collar workers were addressed more often concerning physical exercise and alcohol use, while blue collar workers received more advice concerning all four behaviors.

Patients on sick leave or with a poor self-rated physical health received more questions and advice/help concerning all four health behaviors. Present smokers received more questions and advice concerning their smoking habits than others. Similarly, people dependent on alcohol received more questions and advice about alcohol.

### Most and least common areas of questioning and advice

**Figure 1:**
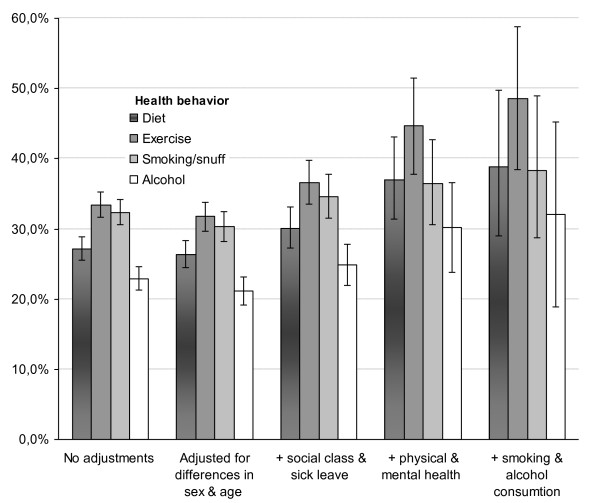
**Percent patients (including 95% CI) who were questioned about their health behavior, adjusted for their background characteristics**.

Figure 1 shows that it was less common to have been asked questions about alcohol habits (as indicated by the comparisons of the 95% CI). Differences between health behaviors remained when different patient characteristics were taken into account. Further adjusting for smoking and alcohol use in addition to patient characteristics possibly reduced the difference between smoking and alcohol somewhat. Smoking no longer differed significantly from alcohol use, even if the tendency was similar. Pseudo R square was between 0.06 and 0.10.

**Figure 2:**
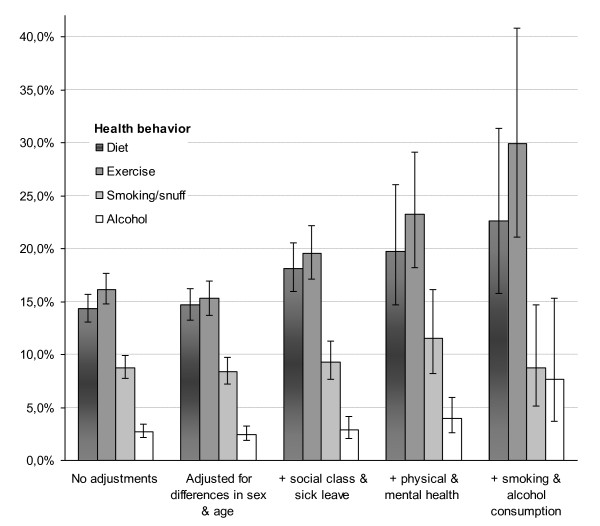
**Percent patients (including 95% CI) who received health behavior advise/help, adjusted for their background characteristics**.

Figure 2 shows that it was less common to have received advise/help concerning alcohol use, even significantly less common than for smoking. This pattern remained, when adjusting for respondents characteristics. However, when smoking and alcohol consumption were included in the model, the percentage indicated for smoking and alcohol use were the same and difference between them non-significant (as indicated by the 95% CI). Thus, in average over all the different categories of the independent variables the likelihood of having received alcohol or smoking advice did not significantly differ from each other. Pseudo R square for smoking and alcohol were 0.50 and 0.39 respectively.

### The association between stage of change and receiving questions/advice concerning alcohol and smoking

Next, the respondents' stage of change were examined, Table 3. The odds for having been asked questions about alcohol use the previous year were significantly higher for persons who reported a decreased alcohol use less than 6 months ago, or 6-12 months ago, than for those who reported no intention to decrease their use. The odds to have received advice/help were similarly higher. The odds were furthermore higher among persons who intended to decrease their alcohol use than among those who did not intend to do so. Even if those who intended to and those who did not intend to decrease their use were collapsed into one reference category - the odds to have received advice were still higher for persons who reported a decreased alcohol use less than 6 months or 6-12 months ago.

**Table 3:** Odds ratio for people in different stages of change1 to have received questions and advice/help to reduce their alcohol consumption or to cease smoking using logistic regression models among patients who had visited health care during past 12 months.

	Does not intend to reduce/cease within 6 months	Intends to reduce/cease within 6 months	Intends to reduce/cease within a month	Reduced/ ceased less than 6 months ago	Reduced/ ceased 6-12 months Ago	Reduced/ ceased more than 12 months ago	
	OR	OR	OR	OR	OR	OR	*n total*
**Reduced alcohol consumption**							
Questions	1.0^4^	1.44	1.61	2.35***	2.13**	1.36*	
*n*	*1433*	*79*	*52*	*97*	*86*	*501*	*2248*
							
Advice/help	1.0^5^	8.84***	18.2***	11.6***	12.0***	2.96**	
Advice/help II	1.0^6^	1.0^6^	1.0^6^	6.29***	6.49***	1.60	
*n*	*1417*	*74*	*52*	*96*	*84*	*487*	*2210*
							
**Smoking cessation**							
Questions	1.0^2^	1.62*	0.58	1.23	0.64	0.49***	
*n*	*292*	*154*	*49*	*55*	*43*	*723*	*1316*
							
Advice/help	1.0^3^	1.63*	0.52	0.76	0.54	0.05***	
*n*	*287*	*155*	*50*	*56*	*44*	*702*	*1294*

* p < 0.05 ** p < 0.01 *** p < 0.001 The significance level is given for each category contrasted against the average of the variable's all categories using Wald chi-square tests in logistic regression models.

^1 ^People who had never smoked were not addressed by the stage of change item and excluded from the modeling. Similarly, persons who drank less than a glass of alcohol during the last twelve months were not addressed by this stage of change item and therefore excluded from the alcohol model.

^2^The OR 1.0 in the reference group corresponds to a likelihood of 45.6 percent of having received questions.

^3 ^The OR 1.0 in the reference group corresponds to a likelihood of 32.1 percent of having received advice/help.

^4 ^The OR 1.0 in the reference group corresponds to a likelihood of 20.1 percent of having received questions.

^5 ^The OR 1.0 in the reference group corresponds to a likelihood of 1.0 percent of having received advice/help.

^6 ^Here the first three categories were collapsed and used as the reference category against which the other categories were contrasted. This was done in order to compare the odds for those who had decreased their consumption against the odds for all those who had not or only intended to decrease their consumption. The OR 1.0 in the collapsed reference group corresponds to a likelihood of 1.8 percent of having received advice/help.

The odds to for having received questions and advice about smoking previously were significantly higher for persons who reported an intention to cease smoking within the next 6 months, than for those who did not.

### The association between questioning and stage of change independently of advice concerning alcohol

Table 4 shows the results from ordered logistic regression models estimating the relative odds to be in a later stage of change. Model 1 shows that being questioned was related to being in a later stage of change independently of advice. Model 2 shows that a younger age was significantly related to being in a later stage of change independently of questions and advice, but gender was not.

**Table 4:** Odds ratios (OR) for those being questioned and having received advice/help concerning their alcohol habits, gender, and age using ordered logistic regressions modeling the five stages of change1 regarding the patients' alcohol habits (n = 1 693).

	Bivariate	Model 1	Model 2	Model 3
	OR	OR	OR	OR
No questions	1.0	1.0	1.0	1.0
Questioned	2.03***	1.47*	1.45*	1.00
				
No advice	1.0	1.0	1.0	1.0
Advised	8.45***	6.43***	6.74***	1.04
				
Age	.956***	-	.956***	.953***
				
Gender				
Female	1.0	-	1.0	1.0
Male	1.08	-	1.04	1.08
				
Interactions				
Questioned*female	-	-	-	1.92*
Advised*male	-	-	-	5.01*
Advised*age	-	-	-	1.038*

* p < 0.05 ** p < 0.01 *** p < 0.001 The significance level is given for each category contrasted against the average of the variable's all categories using Wald chi-square tests in logistic regression models.

^1 ^People who had reduced their alcohol consumption more than 12 months ago were excluded. Similarly, persons who drank less than a glass of alcohol during the last twelve months were not addressed by the stage of change item and are therefore also excluded.

Model 3 in Table 4 includes interaction terms. It shows that women - but not men - who were questioned had higher odds to be in a later stage of change. Moreover, men - but not women - who received advice had higher odds to be in a later stage of change. It also shows that while younger age was related to be a later stage of change in general, among those who received advice this was not the case.

## Discussion

Of the primary care visitors, 23 percent (n = 593) reported being asked about their alcohol habits and 3 percent (n = 69) reported receiving advice or help. Estimates were lower for alcohol than for smoking, physical exercise, and dietary habits - results similar to another Swedish study [4]. However, after adjusting for health behaviors and patient characteristics, there were no significant differences between smoking and alcohol use. In fact 47 percent of the present smokers and 45 percent of those dependent on alcohol reported receiving questions from primary care, while 32 percent of the smokers and 39 percent of those dependent on alcohol reported receiving advice or help. Smoking and alcohol related advice, respectively, reached about the same proportion of the population in need of advice or counseling. This corroborates findings from previous studies [4,5], and it also indicates that over a period of several years a considerable proportion of the smokers and the persons dependent on alcohol in the population is likely to have been reached.

Males, people on long term sick leave, or with a poor physical or mental health more often reported being addressed about their alcohol habits. This pattern was similar for all examined health behavior topics. At the same time, the demographical characteristics of the patients explained less than ten percent of the variance in whether they reported being addressed or not. In spite of this, there was a considerable amount of the target population that reported receiving alcohol related advice. This suggests that the health care personal was quite good at identifying people dependent on alcohol for other reasons, i.e., by other characteristics.

In retrospect, reports of being asked questions were also associated with a decreased alcohol use. This was an association that was independent of the received advice/help for women but not for men.

Thus, simply addressing alcohol habits seems to have been associated with reported decreases of the alcohol consumption. At the same time, both questions and advice were only associated with the intention to cease smoking within 6 months. These results support earlier findings that indicate effects from talk about alcohol at routine health care visits [27]. In two other studies both intervention and control groups showed significant reductions in alcohol consumption when researchers evaluated the effect from brief primary care counseling performed by physicians and nurses in ordinary health care reception [39,40]. Nevertheless, there were no significant difference between controls and the heavy drinkers randomized to receive brief counseling.

In the future it would be interesting to examine the observed association with a more elaborate design. In addition to having groups with only post intervention measures, it would be desirable, for example, to use a quasi-experimental design with randomization and measure before and after the intervention. Effect sizes similar to that of brief counseling have been indicated for the screening procedure [25,26] and it may apparently be considered a separate intervention. It is not known whether the effect from alcohol screening, i.e., using a questionnaire such as AUDIT, differs from being addressed face to face with questions from a physician or a nurse. This would be interesting to know, not least, among the larger population of low risk consumers of alcohol. Differentiating between different forms of interventions, e.g., screening, questions, and advice, would also be helpful in corroborating previous research and in evaluating which measures are most helpful.

One limitation in the present study is that it is not known how the health behavior topics were addressed during the consultations or how the recommended help was provided or conceived (but see [41]). For example, a few of the respondents (n = 5) who received advice or help did not report being asked questions about their alcohol habits. It is possible that talk about alcohol habits is sometimes initiated by patients. While screening is not likely to have been substantially spread, it may have been applied by some primary care centers in the county. The health professionals may also be more or less educated in promoting a healthier life style. There have, for example, been courses for primary care personal advocating the use of Motivational Interviewing (MI) based approaches since the time this study was collected.

The cross-sectional study design and the retrospective self-reported measures is another limitation. Since data on health behavior before or at the time of the intervention is lacking, change could not be estimated for any particular group, such as for those dependent on alcohol. Hence, some of the decrease in alcohol use derived from a decrease among low risk consumers. This might also explain the different results for men and women. Men drank more, which could have explained why they need advice or help rather than questioning. But perhaps women are also more responsive to being addressed.

Another consequence of this design was the ambiguous results concerning the hazardous alcohol users. The percent who were asked questions among them did not differ from the average or the low risk users. But then the AUDIT-score before or at the time of the primary care visit was not known. Therefore, it was not possible to know whether many of many of them had succeeded to change their alcohol habits since their visit, or if they were not successfully reached from primary care as a target group. While the health risks for hazardous drinkers have been recognized for many years, the value of identifying this risk group may not have spread to the primary care sector by the time of survey in 2003. It would be interesting to know whether primary care's ability to detect them has altered since then.

The study's response rate was low and the estimated prevalence of hazardous users (11%) and people dependent on alcohol (2%) was somewhat lower than estimates for the general population in Sweden (14% and 3%) [15]. However, the estimated prevalence of smokers was about the same [16]. The proportion of people who received advice from primary care concerning diet, exercise, smoking, and drinking was also similar to a previous study [4].

The measures used in the study were not, besides AUDIT, established instruments with known properties. Though the items had face validity and while similar items have been in use, it would have been desirable to have had their reliability and validity properly verified in the literature. Patient self-report of the content of physician-office visits is commonly used to assess frequency and content of primary care counseling, but to assess change with such cross-sectional data is less customary. Patients' reports were also collected up to 12 months after the health care visits, which may be questionable. While the data is open to recall and social desirability bias, it may be recognized that poor validity is likely to dilute relationships, if anything. It may also be recognized that the effect of questioning which this study implies cannot be examined with the usual experimental designs which have been used to assess change after counseling since the questioning would equal an effect of the selection procedure. Many brief intervention studies are still very much efficacy-oriented, conducted under very controlled and "ideal" circumstances, which often makes it difficult to translate results into routine health care practice. This study can instead be seen as an "uncontrolled non-randomized trial" in that people are asked about changes in their drinking following routine health care visits.

The different results for smoking and alcohol advice concerning their association with the stage of change do suggest that the observed association was not due to any general response bias. While the change we asked for concerning smoking was more drastic - to completely cease, the reason for the different patterns between alcohol use and smoking are otherwise unclear. One may however note that in Sweden the prevalence of smoking has followed a downward trend [42], while the alcohol consumption followed an opposite trend in the years preceding this study [43,44]. In other words, outflow from the group with unhealthy behavior has been larger than inflow for smoking, while the opposite has been true for alcohol use. Given that there are different selection mechanisms in operation, this might have brought about changes over time in the compositions of the groups with unhealthy behavior. In recent decades the category of smokers may, for example, have become an increasingly select group of hardcore smokers, e.g., consumption patterns or the history of unhealthy behavior may have changed. Whether variables associated with cessation or decrease have changed over time presents an interesting issue for future study.

Screening and brief counseling is a cost-effective preventative measure [12]. The scope for targeting health behaviors in primary health care may be somewhat less extensive than the percentages of people *not *reached by advice and questions might suggest. But even less systematically given advice and questioning in primary care settings appears to be associated with reported change.

## Conflict of interests

The authors declare that they have no competing interests.

## Authors' contributions

KDT and KA conceived the study. KA performed the statistical analysis and wrote the draft of the manuscript. KDT and PA revised it critically for important intellectual content and all authors read and approved the final manuscript.
